# Applicability of Diffuse Ultrasound to Evaluation of the Water Permeability and Chloride Ion Penetrability of Cracked Concrete

**DOI:** 10.3390/s18124156

**Published:** 2018-11-27

**Authors:** Eunjong Ahn, Seongwoo Gwon, Hyunjun Kim, Chanyoung Kim, Sung-Han Sim, Myoungsu Shin

**Affiliations:** School of Urban and Environmental Engineering, Ulsan National Institute of Science and Technology (UNIST), Ulsan 44919, Korea; eunjong@unist.ac.kr (E.A.); ksw4430@unist.ac.kr (S.G.); guswns3@unist.ac.kr (H.K.); kcy931208@unist.ac.kr (C.K.); ssim@unist.ac.kr (S.-H.S.)

**Keywords:** diffuse ultrasound, water permeability, chloride ion penetrability, average crack width

## Abstract

This study aims to explore the applicability of diffuse ultrasound to the evaluation of water permeability and chloride ion penetrability of cracked concrete. Lab-scale experiments were conducted on disk-shaped concrete specimens, each having a different width of a penetrating crack that was generated by splitting tension along the centerline. The average crack width of each specimen was determined using an image binarization technique. The diffuse ultrasound test employed signals in the frequency range of 200 to 440 kHz. The water flow rate was measured using a constant water-head permeability method, and the chloride diffusion coefficient was determined using a modified steady-state migration method. Then, the effects of crack width on the diffusion characteristics of ultrasound (i.e., diffusivity, dissipation), water flow rate, and chloride diffusion coefficient are investigated. The correlations between the water flow rate and diffuse ultrasound parameters, and between the chloride diffusion coefficient and diffuse ultrasound parameters, are examined. The results suggest that diffuse ultrasound is a promising method for assessing the water permeability and chloride ion penetrability of cracked concrete.

## 1. Introduction

Water permeability and chloride ion penetrability are widely used as indices to evaluate the durability of cement-based materials. When cracks occur in concrete structures, permeation of water and penetration of harmful ions (e.g., chloride ion) through the cracks may accelerate corrosion and deterioration of steel reinforcements, which can jeopardize the safety and serviceability of the entire structures [1,2,3]. To enable effective inspection of the location, size, and characteristics of concrete cracks, various ultrasonic methods have been developed, and their applications have been attempted in real structures [4,5,6].

Ultrasonic wave velocity [7], transmission of surface waves [8,9], diffusion of high-frequency waves [10,11,12,13,14,15,16], coda wave interferometry [16,17,18], characteristics of guided waves [19], and nonlinear characteristics of ultrasound [20] have been studied for application to the nondestructive evaluation of defects in concrete. Each ultrasonic test method is based on a unique theoretical concept, and has distinct advantages and disadvantages for different applications [5,21]. Among the available ultrasonic methods, diffuse ultrasound can be applied to monitor and evaluate the process of micro-cracking damage, self-healing [15,16] or volumetric change in concrete [18], and it may also be applicable to evaluating the material properties of concrete (e.g., air-voids, setting time) [22,23,24,25]. Furthermore, Ahn et al. [21] suggested that diffuse ultrasound may have an advantage in the evaluation of concrete durability because the diffusion of ultrasonic waves is sensitive to microscale damages as well as macroscale cracks in concrete. The durability of concrete is commonly evaluated by lab-scale experiments such as water permeability, chloride ion penetration, freeze–thaw, and carbonation tests. Among these, water permeability and chloride ion penetrability are directly influenced by crack width, and thus are chosen as the primary durability indices in this study.

Some researchers focused on water permeation characteristics of concrete in both uncracked and cracked conditions [26,27,28,29,30]. In general, the permeability of water is extremely low without external pressure in uncracked concrete [27], while the amount of water permeation is strongly related to crack width in cracked concrete [28,29,30]. Aldea et al. [28,29] investigated the effects of crack width on water permeability and self-healing performance by comparing water permeability and elastic wave transmission tests. Shin et al. [30] researched on the effects of the type and width of crack on the permeability of cracked concrete.

Other researchers emphasized chloride penetration resistance of concrete [30,31,32,33,34,35] for the development of sustainable concrete structures that have longer service lives. Standardized test methods such as NT Build 355 [36], NT Build 492 [37], and ASTM C1202 [38] have been widely used to evaluate chloride ion diffusion in concrete. Jacobsen et al. [30] studied the effects of crack healing on chloride transport properties in concrete. Oh and Jang [32] suggested a model for predicting the chloride diffusivity of concrete that takes in to account its microstructure. Park et al. [33] proposed an analytical model to estimate chloride ion penetration in cracked concrete. Additionally, Jang et al. [34] determined the threshold crack width that would cause a sudden increase of chloride diffusion in steady-state migration tests.

However, the aforementioned test methods are not applicable in most real structures. Although water permeability and chloride ion penetrability are regarded as primary durability indices for concrete structures, evaluations of them were limited to only lab-scale experiments so far. The development of non-destructive ultrasonic techniques is thus needed for the evaluation of the durability properties of existing concrete in the field, which were scarcely investigated to the authors’ knowledge.

As the first step of meeting the aforementioned needs, this research aims to explore the applicability of diffuse ultrasound to the evaluation of water permeability and chloride ion penetrability of cracked concrete specimens, toward future developments and applications of in-situ structural health monitoring techniques. Many different conditions of cracks may occur in concrete structures under various loads, and no single test configuration can fit to all the different situations. This study focused on slab- or wall-like members having a penetrating crack with a relatively uniform width through the member thickness. Diffuse ultrasound, water permeability, and chloride ion penetration tests were conducted on disk-shaped specimens, each having a penetrating crack along the centerline. The effects of crack width on the diffuse wave parameters (i.e., diffusivity and dissipation coefficients), water flow rate, and chloride diffusion coefficient are investigated. The correlations between the average crack width, durability indices (i.e., water flow rate, chloride diffusion coefficient), and ultrasonic parameters (i.e., diffusivity and dissipation coefficients) are examined.

## 2. Theoretical Background of Diffuse Ultrasound

**Figure 1:**
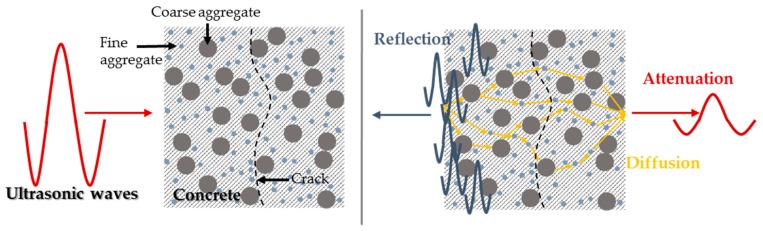
Diffusion phenomena of high-frequency ultrasound in concrete [21].

Several research groups recently pay attention to the diffusion phenomena of high-frequency ultrasound in concrete [10,11,12,13,14,15,16,39]. Most previous ultrasonic non-destructive assessment techniques for concrete structures have been developed through the use of low frequency excitations, typically below 50 kHz. The reason for using the aforementioned low-frequency range is to minimize wave scattering by employing long wavelength components compared to the sizes of heterogeneities (e.g., pores, cracks, fine and coarse aggregates) in concrete [9]. In contrast, when the wavelength of ultrasound is much smaller than such heterogeneities (i.e., high-frequency ultrasound above approximately 200 kHz), coherent parts quickly dissipate and incoherent parts multiply scatter in the medium, which is considered as diffusion phenomena of ultrasound, as illustrated in Figure 1. Therefore, diffuse ultrasound techniques have an advantage for the characterization of microstructural changes of concrete [21].

The diffusion behaviors of ultrasound in concrete can be assumed to follow the governing equations of heat conduction. In this study, the two-dimensional diffusion equation is used to quantify the diffuse ultrasound parameters in the disk-shaped specimens. The governing equation for two-dimensional diffusion problems can be expressed by [10,40]:

(1) $$D\nabla^{2}\langle E\left(x,y,t\right)\rangle -\frac{\partial }{\partial t}\langle E\left(x,y,t\right)\rangle -\sigma \langle E\left(x,y,t\right)\rangle =f\left(x,y,t\right)$$

Here, D and σ represent the diffusivity and dissipation coefficients of ultrasound, respectively. 〈E(x,y,t)〉 stands for the ultrasonic spectral energy density, x and y are the ordinates of the considered location, t is the time, and *f* is the external source. The solution for the diffusion equation in Equation (1) in an infinite medium can be written as follows [10,40]:

(2) $$\langle E\left(x,y,t\right)\rangle =\frac{E_{0}}{4D\pi t}e^{-\frac{x^{2}+y^{2}}{4Dt}-\sigma t}$$

Here, $E_{0}$ is the initial ultrasonic spectral energy density, and $x^{2}$ plus $y^{2}$ represents the square of the distance between the source and receiver. Equation (2) can be rewritten in a logarithmic form for regression analysis to determine the ultrasonic diffusivity and dissipation coefficients using experimental data as follows:

(3) $$\log\langle E\left(x,y,t\right)\rangle =C_{0}-\log\left(Dt\right)-\frac{x^{2}+y^{2}}{4Dt}-\sigma t$$

where, $C_{0}$ is a constant related to the initial energy.

## 3. Test Specimens

In this study, disk-shaped concrete specimens, each with a penetrating crack along the centerline through the thickness, were used to evaluate the diffusion characteristics of ultrasonic waves, water permeability, and chloride ion penetrability of cracked concrete. This section describes the materials, dimensions, and fabrication procedures of the test specimens (Section 3.1), and introduces an image-based method to measure the average crack width in each specimen (Section 3.2).

### 3.1. Preparation of Specimens

The disk-shaped specimens used are 100 mm in diameter and 50 mm in thickness. The mix proportions of concrete are as follows: the water-to-cement ratio is 0.4, the aggregate-to-cement ratio is 2.0, the maximum size of aggregate is 5 mm, which is commonly used in cement-based mortars, and no coarse aggregate is included. When coarse aggregate is used, generated crack faces will be more irregular and uneven in general. Thus, this study first tackles the more fundamental case with no coarse aggregate.

All the specimens were demolded after one day of curing, and then were immersed in water for curing. After 90 days of curing, all the specimens were dried at room temperature of 20–25 °C for at least 7 days to avoid possible influence of the change of internal moisture content and drying shrinkage on the test results.

**Figure 2:**
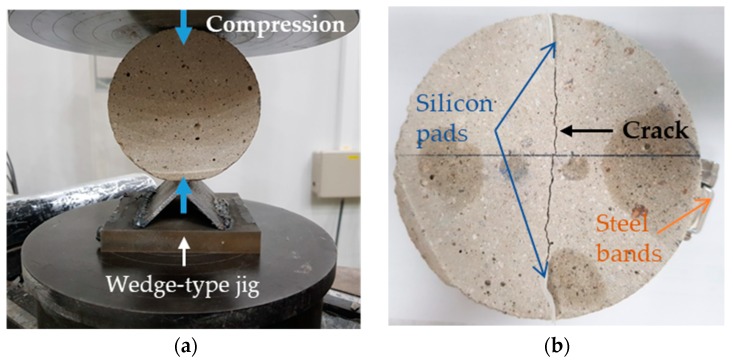
Fabrication of cracked specimens: (**a**) Splitting tension method; (**b**) A specimen with a realistic penetrating crack along the centerline through the thickness.

To fabricate a realistic penetrating crack along the centerline of each disk-shaped specimen, a wedge-type jig shown in Figure 2a was mounted on the bottom plate of a universal testing machine (HDX-1500, INSTRON, Norwood, MA, USA). Then, the specimen was laid on its side and was split into approximately half pieces, by means of the splitting tension method [30]. Silicon pads 50 mm long and 15 mm wide were inserted between the split pieces at the ends to fabricate a crack approximately 70 mm long through the thickness of the specimen. The intended crack width varied from about 0.2 to 0.5 mm. Finally, the split pieces of each specimen with silicon pads inserted were tied together using steel bands along the circumference of the specimen, as shown in Figure 2b [30]. When assembling the two split pieces, the misalignment of crack faces as well as the variation of crack width were minimized by using a microscope, so that the fabricated crack had a relatively uniform width along the entire length and depth.

### 3.2. Determination of Average Crack Width

**Figure 3:**
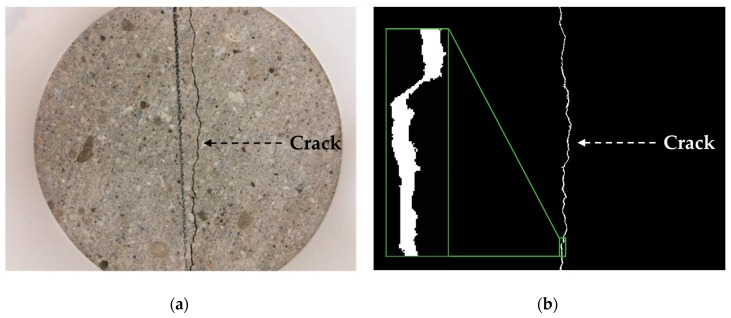
Crack width measurement using image binarization technique: (**a**) Original RGB image; (**b**) Binary image.

Recently, computer vision and image binarization techniques are often employed to not only detect cracks but also measure the dimensions of cracks in concrete structures [41,42]. At first, original RGB images for cracked specimens, as shown in Figure 3a, are converted to grayscale images of up to 256 different levels. Then, the grayscale images are converted to binary images, expressed by 0 (black) and 1 (white) only, as shown in Figure 3b. The pixel values of a binary image are determined considering both threshold and mean values of the pixels located around a particular pixel.

In this study, the average crack width in each specimen was defined as the area of the crack at the flat face divided by the length of the crack. The area of the crack was determined by counting the number of white pixels in the binarized image (Figure 3b), and the length of the crack was determined by connecting the pixels located in the middle of each row along the crack. The image resolution of the camera used is 6800 PPCM (pixel per centimeter), and the focal length is 4.15 mm. The image binarization method proposed by Sauvola et al. [42,43] was used to acquire the binary image, based on comparative analysis for the computational cost and accuracy of crack size estimation by various methods [41]. The technical details of the image binarization method used here are described in the authors’ previous studies [41,42].

## 4. Test Methods

Three independent tests, which are diffuse ultrasound, water permeability, and chloride ion penetration tests, were conducted to determine the diffuse ultrasound parameters (i.e., diffusivity and dissipation), water flow rate, and chloride diffusion coefficient of cracked concrete. The diffuse ultrasound test employed signals in the frequency range of 200 to 440 kHz (Section 4.1). The water permeability test employed a constant water-head method (Section 4.2), and the chloride ion penetration test followed a modified steady-state migration method in accordance with NT Build 355 (Section 4.3).

### 4.1. Diffuse Ultrasound Test

#### 4.1.1. Test Configuration

**Figure 4:**
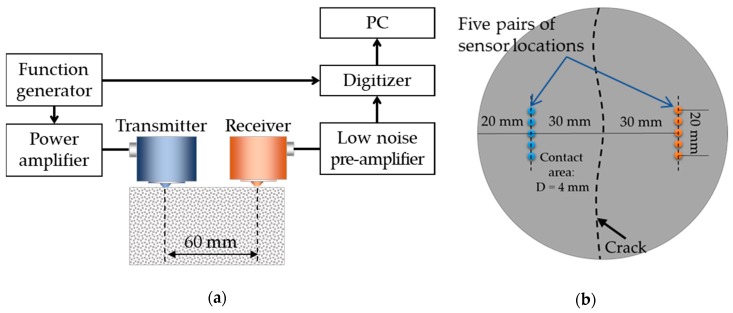
Schematic illustration of the test setup for diffuse ultrasound: (**a**) Side view; (**b**) Top view.

Figure 4a illustrates the test setup used for the measurement of diffuse wave signals. First, 500 kHz pulse signals with a 1 μs duration are produced using an arbitrary waveform generator (33512B, Keysight Technologies, Santa Rosa, CA, USA), and amplified 100 times using a power amplifier (HSA-4014, NF, Yokohama, Japan). A pair of transducers (V101-RB, Panametrics, Waltham, MA, USA) with a 500 kHz center frequency are installed near the center of the specimen (at each of five different locations in turn) as the transmitter and receiver (Figure 4), using vacuum grease. An aluminum cone of 4 mm diameter is attached to the face of each transducer to concentrate the ultrasonic energy input at a small spot and avoid the phase cancellation of short wavelength components due to large-area contacts between the transducers and concrete. The distance between the transmitter and receiver is set to 60 mm, which is deemed adequate to evaluate the diffusion characteristics of high-frequency wave components in concrete [10,11,12,13,14,15].

The received signals are amplified 500 times for crack-free specimens or 5000 times for cracked specimens using a low-noise pre-amplifier (SR560, Stanford Research Systems, Sunnyvale, CA, USA); signal-to-noise ratios were much lower in the cracked specimens. Then, the received analog signals are converted to digital signals using a data acquisition system (Picoscope 4262, Pico Technology, St Neots, UK). Considering appropriate resolutions for the measurement of diffuse wave signals in concrete, a 16-bit digitizer with a 10 MHz sampling rate is used. To improve the signal-to-noise ratio, the diffuse wave measurements are repeated 500 times at each transducer location (with no detachment or reattachment), and the measured raw signals are averaged, which is called “time averaging”.

**Figure 5:**
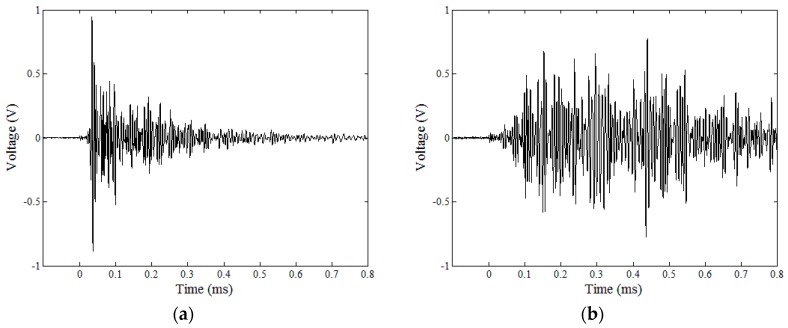
Measured diffuse-wave signals after time averaging: (**a**) No crack (amplified 500 times); (**b**) 0.01 mm width crack (amplified 5000 times).

Examples of time-averaged diffuse-wave signals in crack-free and cracked specimens are shown in Figure 5.

#### 4.1.2. Data Processing

**Figure 6:**
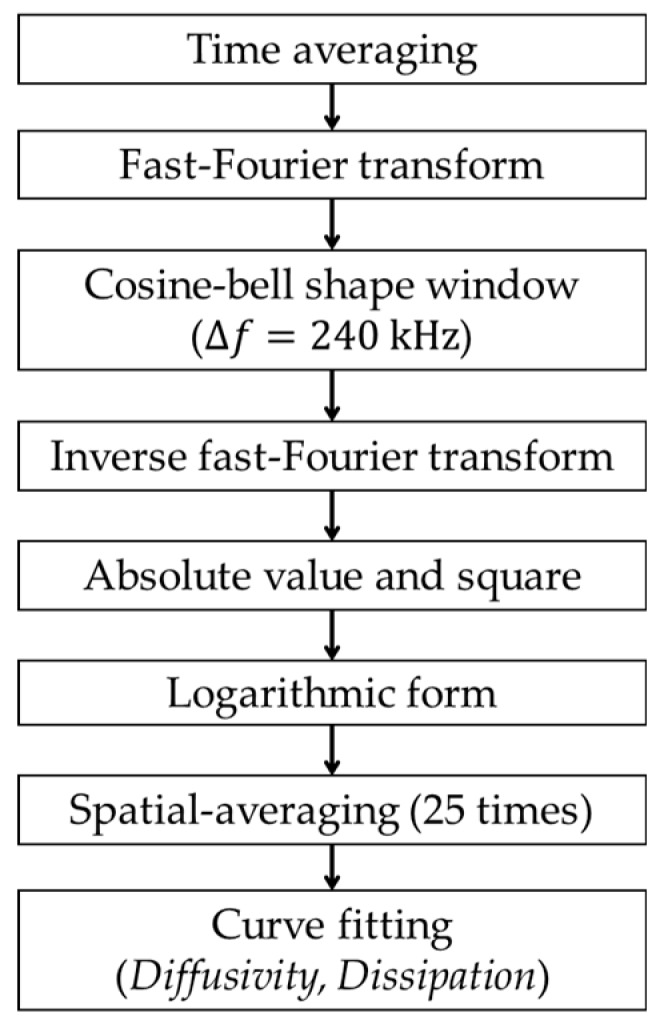
Flow chart of data processing.

**Figure 7:**
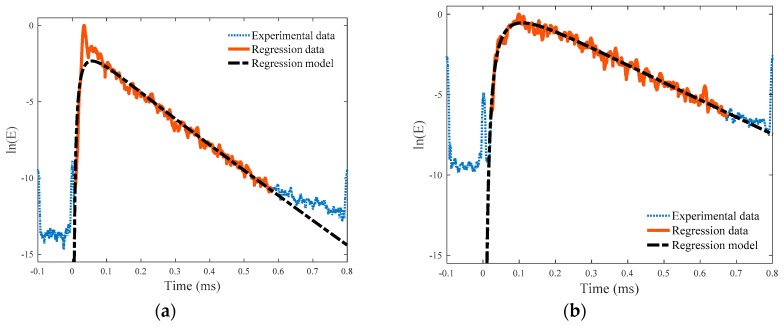
Ultrasonic spectral energy density after spatial averaging: (**a**) No crack; (**b**) 0.01 mm width crack.

Figure 6 describes the flow chart of the data processing. To determine the spectral energy density from the measured time-domain signals, time–frequency analyses are performed for a selected frequency range [40]. First, the time-averaged raw signals in Figure 5 are fast Fourier transformed to the frequency domain, and the transformed data are filtered through a cosine bell-shaped window to isolate data within the frequency range from 200 to 440 kHz, which is selected considering the variation and reliability of diffuse wave signals. For this, the bandwidth of the window in frequency domain is taken as 240 kHz. Then, the windowed signals are inverse fast Fourier transformed to time domain, and the obtained absolute values are squared. The squared values represent the spectral energy densities of diffuse wave signals in the chosen frequency range. Finally, the determined ultrasonic spectral energy densities are plotted on a logarithmic scale (Figure 7) to match with the theoretical solution in Equation (3). The diffuse ultrasound parameters (i.e., diffusivity and dissipation) are determined through regression analysis between the experimental data and theoretical solution of Equation (3) by minimizing the sum of absolute residuals (i.e., least absolute residual method). The regression range for the test data was determined by considering the signal-to-noise ratio, which included approximately 70% of the peak signal level in each case.

Considering the heterogeneous properties of concrete and the effect of sensor installation conditions, the diffuse wave measurements and time–frequency analyses are performed 25 times for each specimen, as explained in the following. A pair of transducers are installed at five different locations within a 10 mm range from the centerline as shown in Figure 4b, and the transducers are detached and reattached five times at each location. Finally, the 25 sets of measured data are averaged, which is called “spatial averaging”.

Figure 7 presents examples of spectral energy density data in the selected frequency range of 200 to 440 kHz for crack-free and cracked specimens; the blue dotted line shows the whole spectral energy density data determined from the spatial averaging, and the red solid line indicates the energy density data used in the regression analysis. Additionally, the black dashed-dotted line indicates the best-fitting curve of the regression analysis.

### 4.2. Water Permeability Test

**Figure 8:**
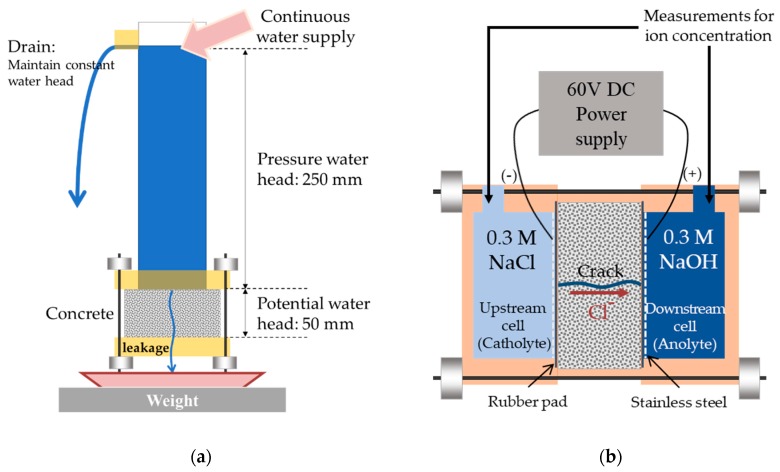
Schematic illustration of water permeability and chloride ion migration test setups: (**a**) Water permeability test; (**b**) Chloride ion migration test.

Both constant-head [30] and falling-head [27,28,29] water permeability tests were applied for lab-scale cracked concrete specimens. In this study, a constant-head test apparatus, shown in Figure 8a, was used to evaluate the water permeability of the disk-shaped specimens with a penetrating crack; the water flow rate was determined by measuring the amount of water that passed through the crack when maintaining a constant water head over a certain duration [44]. Note that this test apparatus was originally designed to investigate factors affecting the water permeability of cracked concrete [30].

The water flow through a cylindrical pipe under a constant pressure can be estimated using Equation (4), which is known as Hagen–Poiseuille’s law, by assuming a laminar flow (Newtonian and incompressible) [45]:

(4) $$Q=\frac{\Delta pD^{4}}{128\mu l}$$

where Q is the water flow rate (m^3^/s), Δp is the differential water pressure (N/m^2^), μ is the viscosity of water (Ns/m^2^), l is the length of the pipe (m), and D is the internal diameter of the cylinder (m).The water flow rate *Q* at a penetrating crack through the thickness of the specimen can be rewritten considering the friction force (second area moment of inertia) as a function of the crack length and crack width as follows [30,45]:

(5) $$Q=\frac{\Delta pbw^{3}}{12\mu l}$$

where b is the crack length (m), w is the crack width (m), and l is the thickness of the specimen (m).

In the test apparatus in Figure 8a, the total water head is 300 mm, comprising 250 mm of the pressure water head and 50 mm of the potential water head (i.e., specimen thickness). The water head level was controlled with a drain installed at a distance of 250 mm from the top surface of the specimen. As for the test procedures, a cracked specimen was first assembled inside the water permeability test apparatus. The total water head was set to and maintained at 300 mm. Then, the water supply continued for 5 min before the initiation of measurement, to ensure a steady water flow state through the specimen. The water supply and drain rates were slow enough to avoid the occurrence of any turbulence flow. Lastly, the amount (i.e., weight) of water flow passing through the penetrating crack was measured at 1 min intervals for 5 min, and the average of the measured data divided by the length of the crack was taken as the water flow rate of the specimen with a certain crack width.

### 4.3. Chloride Ion Penetration Test

In this study, NT Build 355 [36], a steady-state chloride ion diffusion/migration test for cement-based materials, was used to measure the chloride diffusion coefficients of the disk-shaped specimens with various crack widths. Figure 8b illustrates the chloride penetration test setup used in this study. The cathode and anode cells were filled with 270 mL of 0.3 M NaCl solution and 270 mL of 0.3 M NaOH solution, respectively. The disk-shaped cracked concrete specimen was installed between the two electrolyte cells, and the gaps between the acryl cells and concrete were blocked using silicon sealant and rubber bands. The steady-state chloride diffusion coefficient was evaluated using the change of chloride ion concentration with respect to time [34] as:

(6) $$D=\frac{RTL}{zF\Delta E}\frac{V}{A}\frac{1}{c_{1}}\left|\frac{\Delta c_{2}}{\Delta t}\right|$$

where D is the diffusion coefficient ($m^{2}/s$), R is the gas constant (8.3145 J/mol⋅K), T is the absolute ambient temperature (K), L is the thickness of the specimen (m), z is the electrical charge of chloride ions (1 equivalent/mole), F is the Faraday number (96,500 C/equivalent), ΔE is the voltage drop across the specimen (V), V is the volume of the cell (m^3^), A is the sectional area of the specimen disk (m^2^), $c_{1}$ is the chloride ion concentration in the upstream cell (catholyte), $\Delta c_{2}$ is the change of chloride ion concentration in the downstream cell (anolyte) (mmol/L), and Δt is the time duration.

Input voltage of 60 V was supplied, and the voltage drop across the specimen thickness was measured as 40 mV. The ambient temperature was maintained constant (300.5 K) for the whole test process. The effective sectional area considering the area blocked by the cells and rubber bands was about 0.2218 m^2^, and the thickness of concrete was 0.05 m in all cases. The chloride ion concentration was measured once every hour for 6 h both in the upstream cell (catholyte) and downstream cell (anolyte), by collecting several milligrams of electrolyte solution from the middle part of each cell.

The chloride diffusion coefficients of the cracked and uncracked areas of the disk-shaped specimen are greatly different, and may be separately determined using Equation (6). In this study, the chloride ion penetrability in the cracked area is of interest because most of the chloride diffusion occurs through the crack. The chloride diffusion coefficient in the cracked area (i.e., through the crack) may be estimated using the measured test data [34] by:

(7) $$D_{cr}=\frac{D_{eq}A_{tot}-D_{uncr}A_{uncr}}{A_{cr}}$$

where $D_{eq}$ is the diffusion coefficient determined for the considered cracked specimen ($m^{2}/s$), $D_{uncr}$ is the diffusion coefficient determined for the uncracked specimen ($m^{2}/s$), $A_{cr}$ is the crack area ($m^{2}$), $A_{uncr}$ is the uncracked area ($m^{2}$), and $A_{tot}$ is the total sectional area of the disk face ($m^{2}$), equal to $A_{cr}$ plus $A_{uncr}$.

## 5. Experimental Results and Discussion

### 5.1. Effect of Crack Width on Water Permeability

**Figure 9:**
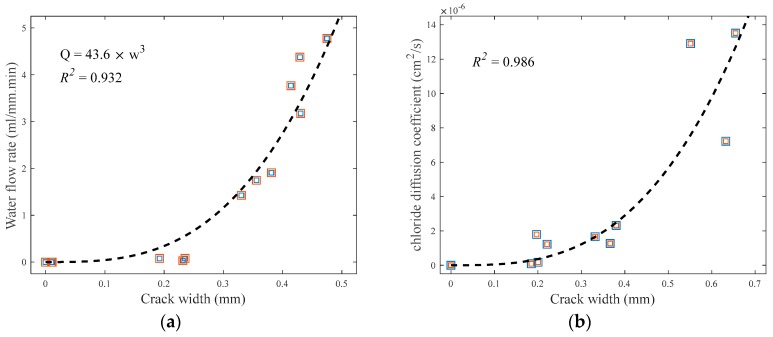
Change of (**a**) water flow rate and (**b**) chloride diffusion coefficient with respect to average crack width.

Figure 9a presents the increase of water flow rate according to the increase of average crack width in the cracked specimens. The experimentally determined water flow rate (*Q*) is well represented by a cubic polynomial function of average crack width based on Equation (5). The regression analysis for the test data in Figure 9a yields the coefficient of the cubic polynomial function as 43.6, and the coefficient of determination (*R*^2^) of the derived best-fit model is 0.932, as noted in the figure.

### 5.2. Effect of Crack Width on Chloride Ion Penetrability

The chloride diffusion coefficient of the uncracked specimen was measured $3.23\times 10^{-10}$ ($m^{2}/s$) in this study, and the coefficient in free water is known to be $2.03\times 10^{-5}$ ($m^{2}/s$). All the determined chloride diffusion coefficients at crack ($D_{cr}$) for different crack widths are within the lower and upper bounds of uncracked concrete and water.

Figure 9b shows the increase of chloride diffusion coefficient in the cracked area ($D_{cr}$) in accordance with the increase of average crack width in the cracked specimens. The chloride diffusion coefficient in the cracked area is calculated using Equation (7). The regression analysis between the measured chloride diffusion coefficient in the cracked area and average crack width results in a cubic function with one parameter. The *R*^2^ value of the best-fitting curve shown in the figure is approximately 0.986. The variation of chloride ion penetrability (Figure 9b) generally increases as the average crack width increases, and is greater than that of water flow rate (Figure 9a).

### 5.3. Effect of Crack Width on Diffuse Ultrasound Parameters

In this study, diffuse wave components in the frequency range of 200 to 440 kHz were selected to examine the diffusion characteristics of ultrasound in cracked concrete. For wave frequencies above 440 kHz, it is difficult to acquire a reliable curve fitting due to low signal-to-noise ratios. On the other end, for wave frequencies below 200 kHz, it is difficult to separate diffuse wave components from coherent waves. Therefore, diffuse wave signals in the frequency range of 200 to 440 kHz are filtered using a cosine bell-shaped window in frequency domain (Figure 6).

**Figure 10:**
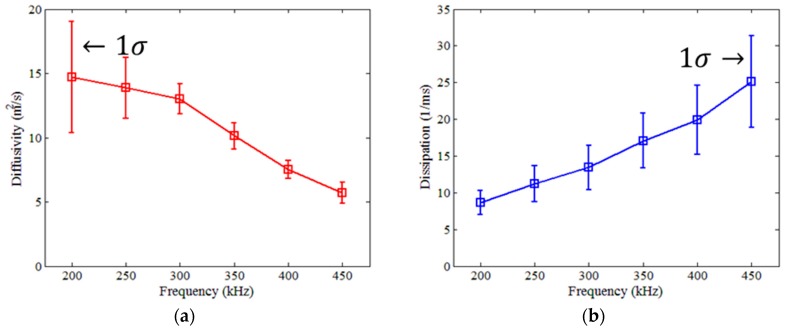
Effects of wave frequency on diffuse ultrasound parameters in crack-free specimens: (**a**) Diffusivity; (**b**) Dissipation.

The effect of wave frequency on the diffusion characteristics of ultrasound in various materials including concrete were discussed in previous studies [12,22,23,24,39,40]. The frequency dependencies and variations of diffuse ultrasound parameters (i.e., diffusivity and dissipation coefficients) for the crack-free specimens of this study are presented in Figure 10; the diffusivity and dissipation coefficients in (a) and (b) respectively are determined at 50 kHz intervals over the range of 200 to 450 kHz. The diffusivity coefficient decreases as the frequency increases, whereas the dissipation coefficient increases as the frequency increases. These correspond to the results from the previous studies [12,22,23,24,39,40]. In addition, the variations of the diffusivity and dissipation coefficients generally increase as the diffusivity and dissipation coefficients themselves increase. The error bars in Figure 10 are plotted using one standard deviation (1-sigma) of the data set obtained from five different crack-free specimens made with the same mix proportions.

To choose a frequency range suitable for the correlation analysis of ultrasonic diffusivity and dissipation coefficients with average crack width, water flow rate, and chloride diffusion coefficient, the average, standard deviation, and coefficient of variation (COV) of both diffusivity and dissipation coefficients are calculated for various frequency ranges as shown in Table 1. The frequency ranges of 300 and 350 kHz show relatively low COV values for the diffusivity coefficient. In contrast, the COV values of the dissipation coefficient are too high for all frequency ranges to be used for further correlation analysis. Therefore, Fourier-transformed diffuse wave signals are filtered using a cosine-bell shaped window (Figure 6) to emphasize the frequency ranges of 300 to 350 kHz.

The cracked specimens show much higher noise levels of the raw diffuse wave data compared with the crack-free specimens due to the presence of discontinuities in the crack gap (Figure 5 and Figure 7). Similarly, fluctuations of the spectral energy density data in the cracked specimens are larger than those in the crack-free specimens, as shown in Figure 7.

**Figure 11:**
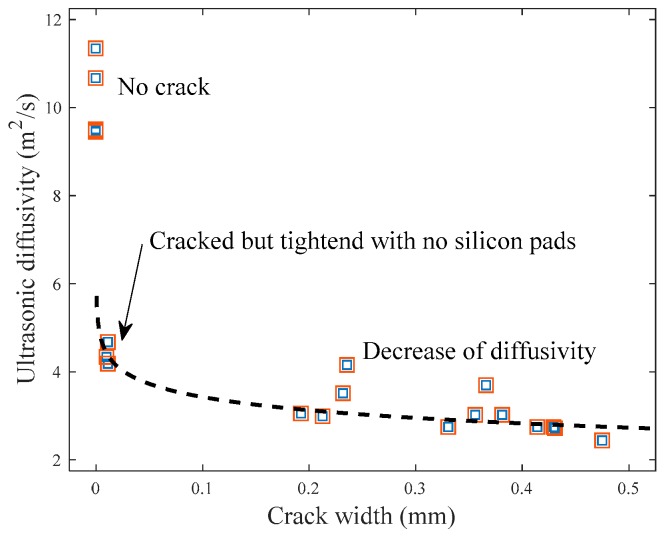
Relationship between ultrasonic diffusivity and average crack width.

Figure 11 presents the relationship between the ultrasonic diffusivity and average crack width. The mean value of the diffusivity coefficient clearly decreases from 10.1 (m^2^/s) in the crack-free specimens to 4.39 (m^2^/s) in the cracked specimens tightened with no silicon pads inserted. Furthermore, an increase in average crack width generally results in a decrease in ultrasonic diffusivity. Previous experimental results agree with the decrease of ultrasonic diffusivity from the increase of crack width [11]. However, the change of diffusivity is not significant in this study as the crack width increases. This is likely because the cracks penetrate through the disk-shaped specimens, which typically is not the case in real structures. A logarithmic model is proposed in Figure 11 to correlate the diffusivity coefficient of ultrasound with the average crack width, and the *R*^2^ value of the best-fitting regression model is approximately 0.788.

### 5.4. Correlation between Ultrasonic Diffusivity and Durability Indices

**Figure 12:**
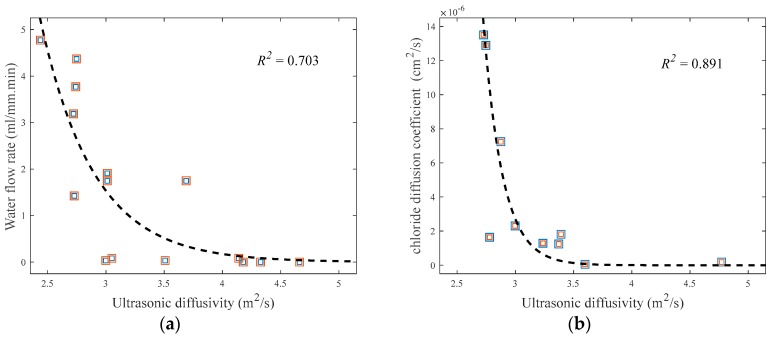
Correlation between the durability properties and ultrasonic diffusivity of cracked concrete: (**a**) Water flow rate; (**b**) Chloride diffusion coefficient.

The effects of average crack width on the water flow rate and chloride diffusion coefficient of cracked concrete are discussed in Section 5.1 and Section 5.2. Further, the most sensitive diffuse-wave parameter (i.e., diffusivity) to the change of average crack width is investigated in Section 5.3. In the following, the correlations between the water flow rate and ultrasonic diffusivity, and between the chloride diffusion coefficient and ultrasonic diffusivity, are examined. Figure 12a shows the relationship between the water flow rate and ultrasonic diffusivity, and Figure 12b displays the relationship between the chloride diffusion coefficient and ultrasonic diffusivity. Both the water flow rate and chloride diffusion coefficient have a decreasing trend as the ultrasonic diffusivity increases, although the variations of both are not negligible. The durability indices rapidly reduce at about 2.5–3.5 m^2^/s of the ultrasonic diffusivity coefficient, which corresponds to the average crack width of approximately 0.3 mm. Exponential functions are adopted for the regression analysis of the measured ultrasonic diffusivity data with the water flow rate and chloride diffusion coefficient data by minimizing the sum of absolute residuals (i.e., least absolute residual method). The *R*^2^ values of the best-fit regression models are approximately 0.703 and 0.891, respectively.

### 5.5. Discussion

The results of this study suggest that the diffuse wave technique is a promising method for the evaluation of the water permeability and chloride ion penetrability of cracked concrete. However, certain technical limitations should be overcome for its practical applications. This section discusses future research needs to overcome the limitations of the proposed methodology, as well as some considerations to improve its reliability:

(1) Presence of coarse aggregate: Mortars with no coarse aggregate were used for both diffuse ultrasound and durability tests to first tackle the more fundamental case, achieving the critical goal of generating a uniform crack width along the length and depth of the crack. Typical concrete contains coarse aggregate of approximately 20 to 25 mm. With the presence of randomly distributed coarse aggregate particles, the crack configuration will be more irregular and uneven, as well as the diffuse wave test results will be affected by different scattering phenomena from the increase of heterogeneity. The effects of coarse aggregate (e.g., size, ratio) should be investigated for practical applications of diffuse ultrasound in concrete structures.
(2) Crack generation method: This study used the tensile splitting and assembling method in fabricating the crack in each specimen. On the other hand, the use of a crack mouth opening displacement measurement device would be more effective to avoid the interlocking issue between paired crack faces. However, a more important issue of this study was to ensure the fabrication of a uniform crack width along the entire length of the crack. Without complete splitting, the crack width typically varies along the length of the crack, which is wider near the center and narrower close to the end. After complete splitting, we used a microscope to minimize the misalignment of crack faces. Accordingly, a more efficient crack generation method should be pursued to not only precisely simulate the actual conditions of real cracks but also resolve the interlocking issue.
(3) Test configuration: The test configuration (e.g., specimen size, crack shape and dimensions) used in the current study may not be appropriate to represent some of real structures. Many different conditions of cracks may occur in concrete structures under various loads, and no single test configuration can fit to all the different situations. This study focused on slab- or wall-like members having a penetrating crack with a relatively uniform width through the member thickness. The test setups used for diffuse ultrasound, water permeability, and chloride penetrability were considered effective for the aforesaid type of cracks even in real structures. Future studies will be needed for other conditions of cracks (e.g., partially closed cracks, those due to bending, multiple cracks) and suitable test configurations.
(4) Specimen dimensions and boundary conditions: An identical size of small specimens were used to conduct both diffuse ultrasound tests and durability tests. High-frequency diffuse ultrasound (e.g., over 200 kHz) tends to quickly spend energy within a relatively small spatial domain. Thus, to apply diffuse ultrasound to the evaluation of voluminous concrete members, a high-energy high-frequency wave generator will be needed. In addition, the diffusion equation for 2-dimensional infinite media was used for evaluating the ultrasonic diffusivity and dissipation without considering the reflections of diffuse wave signals from all boundaries. This approach is useful to investigate qualitative trends of ultrasonic diffusion parameters influenced by the test variables, but may have a limitation in assessing quantitative conditions of real structures with various boundary conditions. Future research is desired to examine the effects of boundary conditions on the diffusion characteristics of ultrasound and the method of taking into account such effects.
(5) Complex material compositions: The diffusion characteristics of a certain frequency range of ultrasound greatly depend on the properties of material compositions in concrete. For instance, the frequency range of ultrasound should be carefully selected considering the dimensions of heterogeneities (e.g., cracks, aggregate particles, pores) to ensure sufficient scattering of the waves. Further studies should be performed to comprehend the effects of material compositions on the behavior of diffuse ultrasound.

## 6. Conclusions

This study examines the feasibility of the application of diffuse ultrasound to the evaluation of durability indices (water permeability and chloride ion penetrability) of cracked concrete. Lab-scale experiments were conducted on disk-shaped concrete specimens, each having a penetrating crack at the centerline. The findings and conclusions of this research can be summarized as follows:

(1) The measured water flow rate in the cracked specimens, defined as the amount of water passing through the crack per minute divided by the length of the crack, was well estimated by a cubic polynomial function of average crack width, which agrees with Hagen–Poiseuille’s law. Similarly, the measured chloride diffusion coefficient was best represented by a cubic function of average crack width.
(2) Both the ultrasonic diffusivity and dissipation in the concrete specimens were significantly affected by the frequencies of chosen wave components. The frequency ranges from 200 to 440 kHz was considered to determine the diffuse ultrasound parameters, based on the variation and reliability of signals.
(3) The diffusion of ultrasound considerably diminished in the presence of the penetrating crack, compared with the crack-free specimens. The diffusivity coefficient generally decreased in accordance with increasing average crack width, although the change of diffusivity was not significant because of the cracks completely penetrating through the specimens.
(4) Both the water flow rate and chloride diffusion coefficient decreased as the ultrasonic diffusivity increased. The two durability indices rapidly reduced at about 2.5–3.5 m^2^/s of the diffusivity coefficient of ultrasound, which corresponds to the average crack width of approximately 0.3 mm.
(5) The test results suggest that diffuse ultrasound is a promising method for the evaluation of the water permeability and chloride ion penetrability of cracked concrete. For its practical use in real structures, however, future research is needed to overcome the limitations of the proposed methodology and improve the accuracy.

## Figures and Tables

**Table 1:** Variance of ultrasonic diffusivity and dissipation according to wave frequency.

Wave Frequency (kHz)	Diffusivity Coefficient (m^2^/s)			Dissipation Coefficient (1/ms)		
	Average	Standard Deviation	COV	Average	Standard Deviation	COV
200	14.7	4.35	29.6%	8.60	1.64	19.0%
250	13.9	2.35	16.9%	11.2	2.44	21.7%
300	13.0	1.16	8.90%	13.5	3.02	22.4%
350	10.1	1.03	10.2%	17.1	3.72	21.8%
400	7.50	0.69	9.20%	19.9	4.74	23.8%
450	5.69	0.81	14.2%	25.1	6.24	24.8%
